# A Facile Synthesis of NiFe-Layered Double Hydroxide and Mixed Metal Oxide with Excellent Microwave Absorption Properties

**DOI:** 10.3390/molecules26165046

**Published:** 2021-08-20

**Authors:** Yi Lu, Pingan Yang, Yanhong Li, Dandan Wen, Jiasai Luo, Shuhui Wang, Fang Wu, Liang Fang, Yu Pang

**Affiliations:** 1 Chongqing Key Laboratory of Photoelectronic Information Sensing and Transmitting Technology, School of Optoelectronic Engineering, Chongqing University of Posts and Telecommunications, Chongqing 400065, China; luyileo@cqupt.edu.cn (Y.L.); liyanhong@cqupt.edu.cn (Y.L.); wendd@cqupt.edu.cn (D.W.); luojs@cqupt.edu.cn (J.L.); wshcqu@yahoo.com (S.W.); 2 School of Automation, Chongqing University of Posts and Telecommunications, Chongqing 400065, China; yangpa@cqupt.edu.cn; 3 State Key Laboratory of Power Transmission Equipment & System Safety and New Technology, Chongqing Key Laboratory of Soft Condensed Matter Physics and Smart Materials, College of Physics, Chongqing University, Chongqing 400044, China; wufang@cqu.edu.cn

**Keywords:** NiFe, layered double hydroxide, mixed metal oxide, electromagnetic wave absorption

## Abstract

Microwave-absorbing materials have attracted increased research interest in recent years because of their core roles in the fields of electromagnetic (EM) pollution precaution and information security. In this paper, microwave-absorbing material NiFe-layered double hydroxide (NiFe-LDH) was synthesized by a simple co-precipitation method and calcined for the fabrication of NiFe-mixed metal oxide (NiFe-MMO). The phase structure and micromorphology of the NiFe-LDH and NiFe-MMO were analyzed, and their microwave-absorbing properties were investigated with a vector network analyzer in 2–18 GHz. Both NiFe-LDH and NiFe-MMO possessed abundant interfaces and a low dielectric constant, which were beneficial to electromagnetic wave absorption, owing to the synergistic effect of multi-relaxation and impedance matching. The optimum reflection loss (*RL*) of NiFe-LDH and NiFe-MMO was −58.8 dB and −64.4 dB, respectively, with the thickness of 4.0 mm in the C band. This work demonstrates that LDH-based materials have a potential application in electromagnetic wave absorption.

## 1. Introduction

With the rapid development of electronic products, electromagnetic (EM) wave irradiation has received increasing attention [1,2]. In order to protect human health and wireless devices from the threat of EM interference, materials with EM wave-absorbing properties were proposed and applied in shielding cases, stealth coatings and microwave darkrooms. As typical microwave absorbers, magnetic metals such as Fe, Co, Ni, Mn and their alloys and oxides, have received considerable attention owing to their high Snoek’s limit, large saturation magnetization, and distinguishable permeability [3]. For example, Fe_3_O_4_ nanocrystals prepared by the hydrothermal method showed a minimum loss value of 21.2 dB at 8.16 GHz [4]. However, the intrinsic drawbacks, such as their high density, low thermal stability and poor oxidation resistance, seriously restrict their further practical applications. Thus, microwave absorbers with strong absorption properties, low density, good thermal stability, a wide absorption frequency range, antioxidant capability, a flexible structure design and facile synthesis routes are required [5,6,7].

According to the electromagnetic wave propagation theory, nanostructured materials with multiple interfaces show great potential as microwave absorbers due to their effective interfacial polarization and associated relaxation loss via surface scattering effects [6,8,9]. In recent years, plenty of special microstructure materials have been developed extensively and their performance in microwave absorption is generally superior [10]. For example, Liu and co-workers prepared Co_20_Ni_80_ hierarchical structures with flower- and urchin-like morphologies by a facile one-step solvothermal treatment, with the minimum RL of −33.5 dB [6]. Yu et al. synthesized flower-like carbonyl iron powder microcrystals, wherein the minimum RL reached as high as −35.5 dB [11]. These excellent EM wave absorption properties were attributed to the surface architectures and hierarchical structures.

Layered double hydroxides (LDHs), also known as hydrotalcite-like materials, are a class of synthetic two-dimensional nanostructured anionic clays, which consist of positively charged hydroxide layers bonded with negatively charged anions in the interlayer space [12,13]. LDHs have a general formula [M^2+^_1−X_M^3+^_X_(OH)_2_]^X+^(A^n−^)_X/n_∙mH_2_O, where M^2+^ and M^3+^ are di- and trivalent cations, respectively, including Mg^2+^, Fe^2+^, Co^2+^, Ni^2+^, or Zn^2+^ and Al^3+^, Mn^3+^, or Fe^3+^, respectively; the value of the coefficient x is equal to the molar ratio of M^2+^/(M^2+^ + M^3+^), and A^n-^ is an anion, such as CO_3_^2−^, SO_4_^2−^, NO_3_^−^, Cl^−^, or PO_4_^3−^ [14,15]. LDHs possess not only stable layered structures but also high specific surface areas and rich inter-layer interfaces. Moreover, the low electronic conductivity is beneficial to their EM absorption in terms of the impedance match mechanism. These properties make LDHs very suitable as EM absorbers. Xu et al. synthesized a NiAl-LDH/graphene nanocomposite, which showed excellent anticorrosive and microwave absorption performance (−41.5 dB at 17.8 GHz) [16]. The calcination of LDHs affords mixed metal oxides (MMOs), which contain spinel and oxide of divalent metal. As mentioned in other articles, oxide nanomaterials have a wide range of responses to electromagnetic radiation due to the functional capacity of the metal center bonded by the oxygen anion, as well as the special structure determined by the crystalline lattice of the material [17]. To the best of our knowledge, LDHs and their calcination productions as microwave absorbers have rarely been reported. Hu et al. constructed NiCo alloy nanoparticles embedded g-C_3_N_4_ hollow microtubular (NiCo-alloy@tubular-g-C_3_N_4_) by the direct pyrolysis of NiCo-LDH@tubular-cyanuric acidmelamine precursors. The obtained NiCo-alloy@tubular-g-C_3_N_4_ achieved a minimum RL value of −35.63 dB at 5.00 GHz [18]. Zhang and co-workers prepared MgFe-layered double oxide/carbon nanohelix composites with a minimum RL of −35.0 dB [9]. However, due to its absence of magnetic properties, the presence of MgO was adverse for EM adsorption.

Nickel, iron, and their compounds are widely used as microwave-absorbing material. The analysis of their electromagnetic properties is of significance to their applications. In this paper, we prepared NiFe-LDH and NiFe-MMO by a mild and simple process. The unique structure with special electromagnetic properties and microwave-absorbing performance was investigated in the frequency range of 2.0–18.0 GHz. The dissipation mechanism for microwaves of the composites were also discussed.

## 2. Results and Discussion

**Figure 1:**
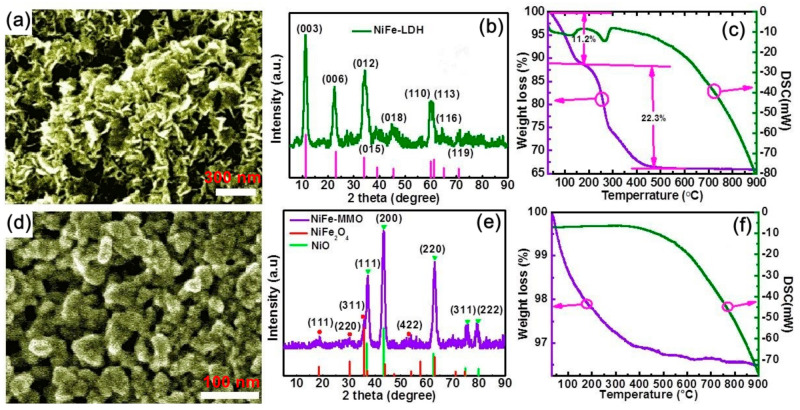
SEM image, XRD patterns and TGA-DSC curves of NiFe-LDH (**a**–**c**) and NiFe-MMO (**d**–**f**).

The phase structure and micromorphology of the nano materials are measured and shown in Figure 1. As shown in Figure 1a and Figure S1, the NiFe-LDH consists of thin platelets and coacervates with a thinness of less than 50 nm, which is the typical product by the co-precipitation method. The random aggregation of the nanoplates forms abundant interfaces which facilitate to the electromagnetic wave incident and reflection. The X-ray diffraction (XRD) patterns reveal that the nanosheets are isostructural with the hydrotalcite-like LDH materials (JCPDS: 40-0215), where the typical diffraction peaks are located at 11.4°, 22.9° and 34.4° corresponding to the (003), (006), (012) planes of NiFe-LDH (Figure 1b). The interplanar spacing is 7.8 Å, which can be calculated from the (003) plane [19]. An additional elemental analysis by inductive coupled plasma emission spectrometer shows that the molar ratio of Ni/Fe is 3.79, corresponding to the molar ratio of M^2+^/M^3+^ of 2–4. This was further confirmed by an X-ray energy dispersive spectrometer (EDS), as shown in Figure S2. The thermal behavior analysis indicates that two steps of weight loss occurred during the calcination process (Figure 1c). The first step exists from 50 to 175 °C with a weight loss of 11.2%, corresponding to the disappearance of interlayer water and absorbed water molecules. The second step ranges from 175 to 475 °C with the mass loss about of 22.3%, corresponding to the removal of the interlayer anions and dehydroxylation of the layers, which means the thermal decomposition and recrystallization of LDH [20]. As the temperature increases, the weight of the sample remains the same, and there is no obvious endothermic/exothermic peak, indicating that the structure of the material achieves relative stability.

When the NiFe-LDH was heated at 600 °C for 2 h, the morphologies and crystal structure are drastically different. As shown in Figure 1d and Figure S3, the calcinated NiFe-LDH is composed of nanoparticles with the diameters of about 30 nm. The nanoparticles agglomerate and induce different voids. Figure 1e shows that the nanoparticles are a composite oxide phase with NiO and NiFe_2_O_4_ structure (namely mixed metal oxides, MMO). The XRD patterns at the peak positions of 2θ = 37.3°, 43.4° and 62.9° correspond to the (111), (200) and (220) planes of NiO (JCPDS: 04-0835), while the characteristic diffraction peaks at 18.4°, 30.3° and 35.7° index to the (111), (220) and (311) planes of NiFe_2_O_4_ (JCPDS: 10-0325). The peak intensities of NiO are higher than that of NiFe_2_O_4_, which means better crystallinity and more NiO in the NiFe-MMO. This is owing to the high molar ratio (3.79) of Ni/Fe in NiFe-LDH precursor. The TGA (Figure 1f) shows that the product upon heating has a smaller weight loss (~2.5%) than the NiFe-LDH (~35%), indicating the volatilization of adsorbed water in the structure.

For the purpose of microwave absorption property measurements, the products (40 wt%) were mixed with paraffin (60 wt%) and compressed to ring shapes with an outer diameter of 7.0 mm, inner diameter of 3.0 mm and thickness of 3.0 mm. An Agilent 85071E vector network analyzer with the Agilent 85055-60001 50 Ohm coaxial air cable were applied to measure the relative permeability and relative permittivity in the frequency range of 2–18 GHz.

**Figure 2:**
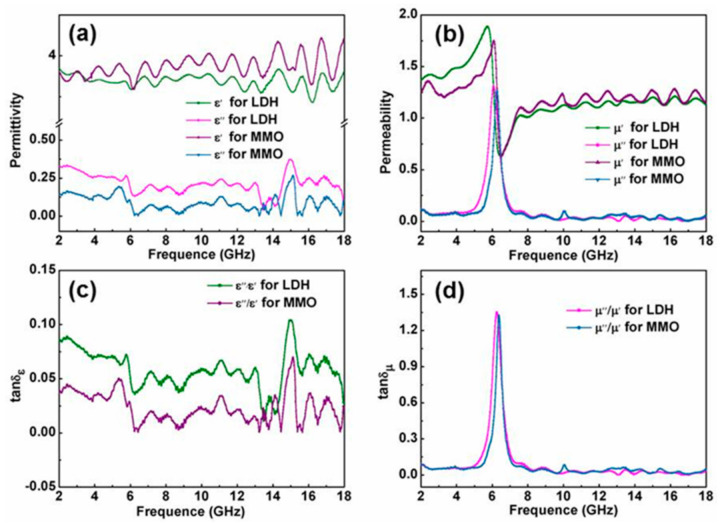
Frequency dependence of complex permittivity (**a**), complex permeability (**b**), dielectric loss tangents (**c**) and magnetic loss tangents (**d**) of the NiFe-LDH and NiFe-MMO.

As shown in Figure 2, the permittivity (ε′ and ε″), permeability (*μ*′ and *μ*″), dieletric loss tangent (tan δε) and magnetic loss tangent (tan δ*μ*) of the NiFe-LDH and the NiFe-MMO show similar trends with frequency. Figure 2a indicates that both the real part (ε′) and imaginary part (ε″) of the samples tend to keep constant over 2–18 GHz. The real parts of the permittivity for LDH and MMO are almost 3.6 and 3.8, respectively, which are lower than that of other NiFe-based materials, such as NiFe_2_O_4_ (4–6) [21], mesoporous NiFe_2_O_4_ (4–4.7) [22] and NiFe Alloy/MWCNT/Epoxy(~5) [23]. The low permittivity is facile for the impedance matching, allowing a greater proportion of microwave incident into the absorbent, which is in favor of electromagnetic wave absorption. The relative permittivity properties of the absorbent depend on electronic, ionic, space-charge, orientational and dipole polarization [24]. However, the dipolar polarization and space-charge polarization (interfacial polarization) are more frequency-dependent in metal-based mixtures materials (metal filler and insulating matrix) [25]. The electronic exchange between Fe^3+^ and Ni^2+^ ions in LDH and MMO induce plenty of space charge and polarization charge under alternating electromagnetic field. Thus, the real part of the permittivity shows a small fluctuation in high frequency.

Generally, the real parts (ε′ and *μ*′) represent the storage capability of electric and magnetic energy, whereas the imaginary parts (ε″ and *μ*″) stand for the inner dissipation abilities, respectively [26]. The real part of permittivity in NiFe-LDH is smaller than that of the NiFe-MMO, but the imaginary part is larger, which means that NiFe-LDH has weaker storage capability of electric and stronger dielectric loss than NiFe-MMO. It is reported that the dielectric loss can be attributed to the lags of polarization between the interfaces and the lamellar LDHs, which have more complicated interfaces than nanoparticles. 

Figure 2b shows the frequency dependence of the real part (*μ*′) and imaginary part (*μ*″) of complex permeability of the NiFe-LDH and NiFe-MMO. At a low frequency, the *μ*′ value of the NiFe-LDH increases quickly from 1.37 at 2.0 GHz to 1.89 at 5.72 GHz, which is higher than that of the NiFe-MMO. It is interesting to find that the NiFe-LDH has a smaller *μ*′ value in the frequency range of 8–18 GHz. For the imaginary part of complex permeability, the NiFe-LDH and NiFe-MMO exhibit almost the same *μ*″ values in the frequency range of 2–5 GHz and 8–18 GHz. However, one major peak occurs at 6.08 GHz for NiFe-LDH and 6.28 GHz for NiFe-MMO, respectively, indicating significantly magnetic loss at this frequencies range.

In addition, the dielectric loss tangents (tan δε = ε″/ε′) and magnetic loss tangents (tan δ*μ* = *μ*″/*μ*′) were also calculated. As presented in Figure 2c,d the tan δε of the NiFe-LDH and NiFe-MMO show a similar minute fluctuation; however, the tan δε values of the NiFe-LDH are larger than that of NiFe-MMO in the range of 2–18 GHz, implying that the NiFe-LDH has better dielectric loss. Compared with the frequency dependence of *μ*, the tan δ*μ* values of both the NiFe-LDH and the NiFe-MMO have a similar trend with the frequency. The magnetic loss tangents (about 1.4 in Figure 2d) in the frequency of 5–7 GHz are larger than the dielectric loss tangents (less than 0.1 in Figure 2c), which means that the magnetic loss of LDH and MMO are dominant compared to the dielectric loss. However, both the two adsorbents are equipped with limited magnetic loss in other frequencies in the range of 2–18 GHz.

**Figure 3:**
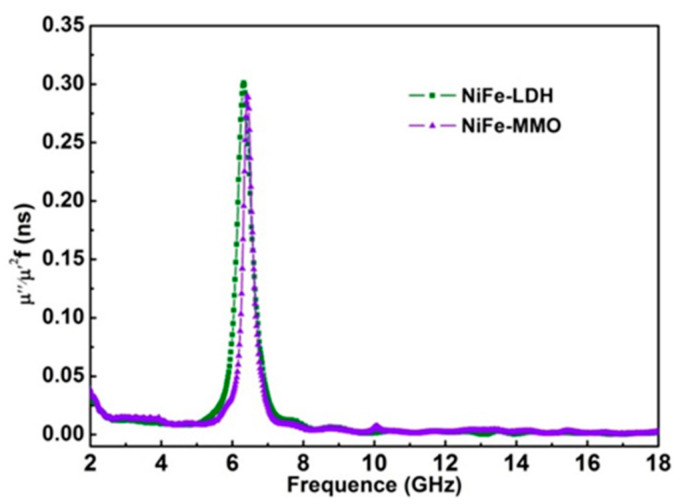
Values of *μʺ*(*μʹ*)^−2^ *f* ^−1^ for NiFe-LDH and NiFe-MMO in the frequency range 2–18 GHz.

The microwave magnetic loss is mainly allocated with several factors, namely, domain-wall displacement, magnetic hysteresis, eddy current loss, and natural resonance [27]. Domain wall displacement loss is negligible since it usually exhibits in the low frequency about 1–100 MHz [24]. Hysteresis comes from irreversible magnetization and can be ignored in a weak applied field [28]. Eddy current loss is related to the diameter of nanoparticles (*d*) and electric conductivity (*σ*), which can be approximately expressed by *μʺ ≈ 2πμ*_0_(*μʹ*)^2^*σd*^2^*f*/*3* [21,29], and here, *μ*_0_ is the permeability of vacuum. If magnetic loss only results from the eddy current loss, the values of *C*_0_ *= 2πμ*_0_*σd*^2^/3 *= μʺ*(*μʹ*)^−2^*f* ^−1^ should be constant as the frequency changes [30]. As is demonstrated in Figure 3, the values of *C_0_* remain approximately unchanged in 2.5–5 GHz and 8–18 GHz, which means the eddy current losses exist in these frequencies. However, as can be seen, *C*_0_ changes drastically in the frequency range of 5–8 GHz. Therefore, it is credible to conclude that the microwave magnetic losses of NiFe-LDH and NiFe-MMO at 6 GHz are mainly ascribed to natural resonance.

Based on the obtained data of complex permittivity and permeability, the *RL* can usually be evaluated by the following equation [31,32,33]:

(1) $$sRL=20\;log\left|\left(Z_{in}-\right.\left.Z_{0}\right)/\left(Z_{in}+\right.\left.Z_{0}\right)\right|$$

(2) $$Z_{in}=Z_{0}\sqrt{\frac{\mu_{r}}{\varepsilon_{r}}}\tanh\left(\left.j\left(\left.\frac{2\pi }{c}\right)fd\sqrt{\mu_{r}\varepsilon_{r}}\right.\right)\right.$$

where $Z_{0}$ is the impedance of free space, $Z_{in}$ is the normalized input impedance between free space and material interface, *c* is the velocity of light, f  is the frequency of microwaves, and d is the thickness of absorbing materials.

**Figure 4:**
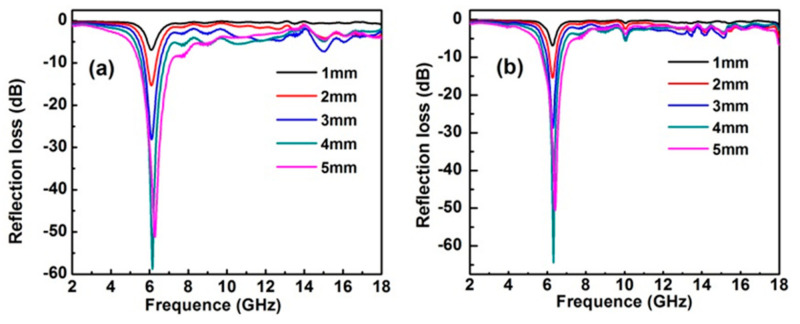
Frequency dependence of reflection loss of the NiFe-LDH (**a**) and NiFe-MMO (**b**) by varying the thickness of the absorbent.

Figure 4 shows the calculated *RL* curves at different specimen thicknesses in the frequency range of 2–18 GHz. The frequencies corresponding to the minimum *RL* values of absorption peaks are 6.14 GHz (LDH) and 6.32 GHz (MMO). Simultaneously, their minimum *RL* values are −58.79 dB (LDH) and -64.43 dB (MMO), respectively. Usually, an *RL* less than −10, −20 dB is equivalent to 90% and 99% of microwave absorption, respectively. For comparison, the minimum *RL* of some microwave-absorbing materials are listed in Table 1. It can be seen that the minimum *RL* of the materials for this work are better than those of the similar microwave-absorbing materials, which implies that the as-prepared NiFe-LDH and NiFe-MMO can be potentially used in the microwave-absorbing field. However, absorption bandwidths (<−10 dB) for NiFe-LDH and MMO are narrow compared with other absorbers. Thus, it is necessary to modify the NiFe-LDH/MMO with other materials, such as graphene, carbon nanohelix or SiO_2_ for a wide absorption frequency band.

The reasons for the excellent microwave absorption properties of the NiFe-LDH and NiFe-MMO are analyzed as follows: firstly, the lower permittivities than other NiFe-based materials are facile for a better impedance matching, allowing a greater proportion of EM incidence into the absorbent, which is an important prerequisite for EM wave absorption. Secondly, the layered nanostructure and random distribution nanosheets of NiFe-LDH may form a discontinuous network, which can induce the EM wave to penetrate the composites. The EM wave will be consumed until its complete disappearance from the discontinuous network. Similarly, porous nanostructures from the stacked nanoparticles in NiFe-MMO can lead to multiple reflections and scatter for energy dissipation. In addition, the nanoporous structures create a large number of interfaces, which enhance the interfacial electric polarization, and are advantageous to the absorption of EM wave. The interfacial multipolar of composites in this study mainly comes from the boundaries between nanosheets in NiFe-LDH, nanoparticles in NiFe-MMO, air bubbles and paraffin matrix. Moreover, the excellent reflection loss in the C band is attributed to the magnetic loss, which weakens the contribution of the thickness cancellation. The microwave magnetic losses of NiFe-LDH and NiFe-MMO at 6 GHz are mainly ascribed to natural resonance.

## 3. Materials and Methods

The NiFe-LDH was synthesized by a co-precipitation method. Specifically, 20.0 mL of metal salt solution containing 4.653 g Ni(NO_3_)_2_∙6H_2_O and 1.616 g Fe(NO_3_)_3_∙9H_2_O (99%, Aladdi, Shanghai, China) were added into a vigorously stirred ammonia (25–28%, Aladdi, Shanghai, China) solution (100.0 mL, 0.5 M) drop by drop. The obtained suspension was stirred mechanically for a further 18 h at 65 °C, then centrifuged and washed thoroughly with deionized water until it had a pH close to 7, and finally dried overnight at 60 °C under vacuum. The calcinated NiFe-LDH was prepared by heating NiFe-LDH in 600 °C for 2 h in a horizontal furnace under the Ar atmosphere.

The morphologies of the obtained products were taken with a JEOL JSM-7800F scanning electron microscope (SEM). The crystal structure was determined using X-ray diffraction (XRD) with Cu Ka radiation, and the thermogravimetry (TGA) and differential scanning calorimetry (DSC) were recorded on a thermal analyzer (STA449F3, Netzsch, Germany) with a heating rate of 10 °C/min in a flowing mixed gas of N_2_.

## 4. Conclusions

In summary, NiFe-LDH and NiFe-MMO are employed for EM wave adsorption. The NiFe-LDH is synthesized by a simple co-precipitation method, and consists of sheet structure, while NiFe-MMO is obtained by the calcination of NiFe-LDH precursor and is composed of nanoparticles. The porous adsorbents exhibit abundant interfaces, which can lead to multiple reflections and scatter for energy dissipation. In comparison with other NiFe-based materials, the lower dielectric constant of NiFe-LDH (~3.6) and MMO (~3.8) devote a better impedance matching. The reflection loss of -58.8 dB for NiFe-LDH and -64.4 dB for NiFe-MMO are observed at the C band with a thickness of 4.0 mm. This study confirms that NiFe-LDH and NiFe-MMO have promising prospects as microwave-absorbing materials.

## Figures and Tables

**Table 1:** Microwave absorption performance of absorbers with NiFe-based materials.

Samples	*RL_min_* (dB)	*d_min_* (mm)	*F_min_* (GHz)	Reference
Ni/Ni(OH)_2_	−25.3	2.0	9.6	[34]
Graphene@Fe_3_O_4_@SiO_2_@NiO	−51.5	1.8	14.6	[35]
NiFe_2_O_4_	−27.5	4.0	~4.5	[36]
NiFe_2_O_4_/PPY	−42.0	3.5	8.0	[37]
NiFe_2_O_4_/rGO	−46.8	4.8	6.8	[38]
NiFe Alloy/MWCNT/Epoxy	−19.0	4.0	8.2	[23]
MgFe-LDO/carbon nanohelix	−35.0	3.0	~11.9	[9]
MgFe-LDO	−7.0	4.0	11.2	[9]
NiFe-LDH	−58.8	4.0	6.1	This work
NiFe-MMO	−64.4	4.0	6.3	This work

PPY: polypyrrole; rGO: reduced graphite oxide; MWCNT: multi-walled carbon nanotube.

## Data Availability

Data of the compounds are available from the authors.

## Supplementary Materials

The following are available online. Figure S1. SEM image of NiFe-LDH with different magnifications. Figure S2. The elemental analysis of NiFe-LDH by EDS. Figure S3. SEM image of NiFe-MMO with different magnifications.

## Sample Availability

Samples of the compounds NiFe-LDH and NiFe-MMO are available from the authors upon request.
