# Supplementary material for: A Facile Synthesis of NiFe-Layered Double Hydroxide and Mixed Metal Oxide with Excellent Microwave Absorption Properties
Source: Molecules. 2021 Aug 20;26(16):5046. doi: 10.3390/molecules26165046 (PMC8398574; doi:10.3390/molecules26165046)
Supplement: Supplementary file 1 [file molecules-26-05046-s001.zip › molecules-1320169-supplementary.pdf]

A Facile Synthesis of NiFe Layered Double Hydroxide and Mixed Metal Oxide with Excellent Microwave Absorption Properties

Yi Lu [1], Pingan Yang [2], Yanhong Li [1], Dandan Wen [1], Jiasai Luo [1], Shuhui Wang [1], Fang Wu[3], Liang Fang [3],* and Yu Pang [1],*

[1] Chongqing Key Laboratory of Photoelectronic Information Sensing and Transmitting Technology, School of Optoelectronic Engineering, Chongqing University of Posts and Telecommunications, Chongqing 400065, P. R. China; luyileo@cqupt.edu.cn(Y. L.); liyanhong@cqupt.edu.cn(Y. L.); wendd@cqupt.edu.cn(D. W.); luojs@cqupt.edu.cn(J. L.); wshcqu@yahoo.com(S. W.);

[2] School of Automation,Chongqing University of Posts and Telecommunications, Chongqing 400065, P. R. China; yangpa@cqupt.edu.cn(P. Y.)

[3] State Key Laboratory of Power Transmission Equipment & System Safety and New Technology, Chongqing Key Laboratory of Soft Condensed Matter Physics and Smart Materials, College of Physics, Chongqing University, Chongqing,, 400044, P. R. China. wufang@cqu.edu.cn(F. W.);

* Correspondence: lfang@cqu.edu.cn(L. F.); Tel.: +86-23-65678362; pangyu@cqupt.edu.cn(Y. P.); Tel.:+86-23-62460522

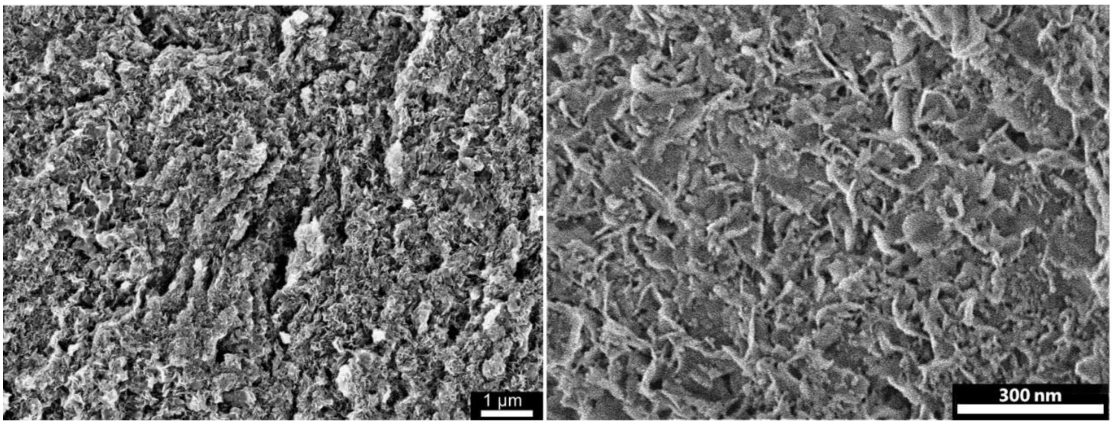

Figure S1. SEM image of NiFe-LDH with different magnifications.

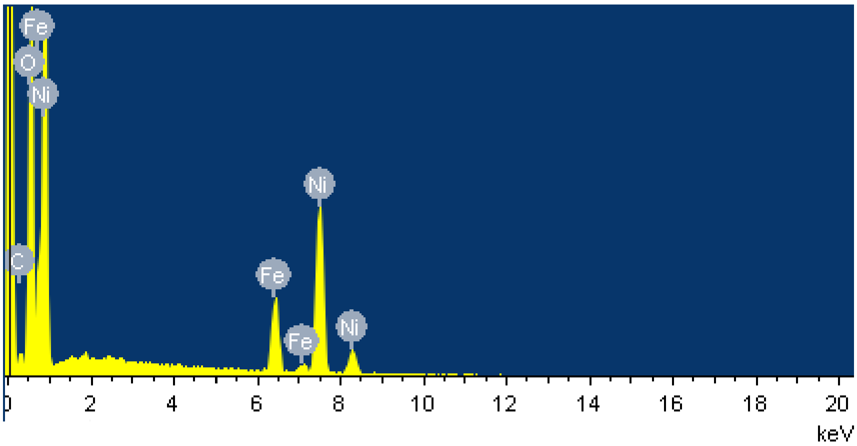

Element	Weight percentage	Atomic percent
C K	8.19	16.85
O K	39.45	60.90
Fe K	10.25	4.53
Ni K	42.12	17.72
Total	100.00	100

Figure S2. The elemental analysis of NiFe-LDH by EDS.

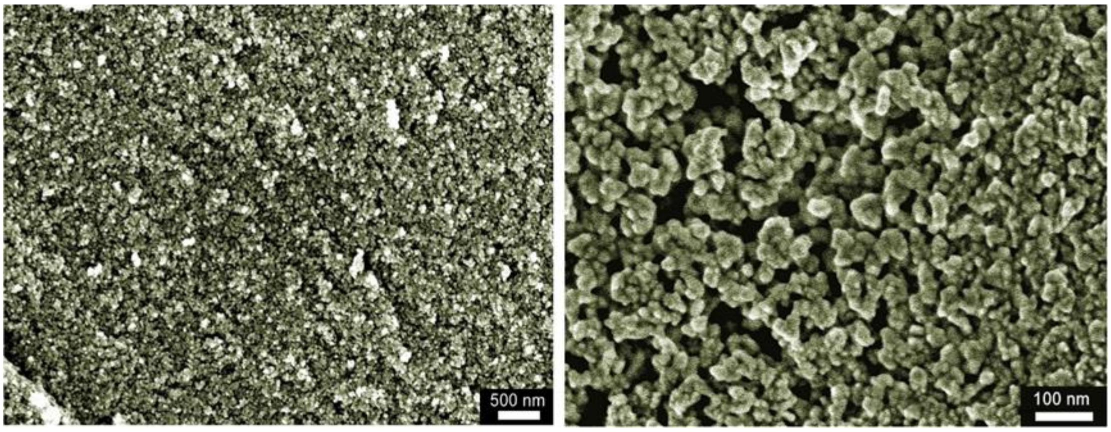

Figure S3. SEM image of NiFe-MMO with different magnifications.
